# Malnutrition leads to increased inflammation and expression of tuberculosis risk signatures in recently exposed household contacts of pulmonary tuberculosis

**DOI:** 10.3389/fimmu.2022.1011166

**Published:** 2022-09-28

**Authors:** Arthur VanValkenburg, Vaishnavi Kaipilyawar, Sonali Sarkar, Subitha Lakshminarayanan, Chelsie Cintron, Senbagavalli Prakash Babu, Selby Knudsen, Noyal Mariya Joseph, C. Robert Horsburgh, Pranay Sinha, Jerrold J. Ellner, Prakash Babu Narasimhan, W. Evan Johnson, Natasha S. Hochberg, Padmini Salgame

**Affiliations:** ^1^ Division of Computational Biomedicine, Boston University School of Medicine, Boston, MA, United States; ^2^ Bioinformatics Program, Boston University, Boston, MA, United States; ^3^ Department of Medicine, Center for Emerging Pathogens, Rutgers-New Jersey Medical School, Newark, NJ, United States; ^4^ Department of Preventive and Social Medicine, Jawaharlal Institute of Postgraduate Medical Education and Research, Puducherry, India; ^5^ Department of Medicine, Boston Medical Center, Boston, MA, United States; ^6^ Department of Microbiology, Jawaharlal Institute of Postgraduate Medical Education and Research, Puducherry, India; ^7^ Section of Infectious Diseases, Boston University School of Medicine, Boston, MA, United States; ^8^ Department of Epidemiology, Boston University School of Public Health, Boston, MA, United States; ^9^ Department of Clinical Immunology, Jawaharlal Institute of Postgraduate Medical Education and Research, Puducherry, India

**Keywords:** tuberculosis, malnutrition, TB biomarkers, inflammation, immunoregulation

## Abstract

**Background:**

Most individuals exposed to *Mycobacterium tuberculosis* (Mtb) develop latent tuberculosis infection (LTBI) and remain at risk for progressing to active tuberculosis disease (TB). Malnutrition is an important risk factor driving progression from LTBI to TB. However, the performance of blood-based TB risk signatures in malnourished individuals with LTBI remains unexplored. The aim of this study was to determine if malnourished and control individuals had differences in gene expression, immune pathways and TB risk signatures.

**Methods:**

We utilized data from 50 tuberculin skin test positive household contacts of persons with TB - 18 malnourished participants (body mass index [BMI] < 18.5 kg/m2) and 32 controls (individuals with BMI ≥ 18.5 kg/m2). Whole blood RNA-sequencing was conducted to identify differentially expressed genes (DEGs). Ingenuity Pathway Analysis was applied to the DEGs to identify top canonical pathways and gene regulators. Gene enrichment methods were then employed to score the performance of published gene signatures associated with progression from LTBI to TB.

**Results:**

Malnourished individuals had increased activation of inflammatory pathways, including pathways involved in neutrophil activation, T-cell activation and proinflammatory IL-1 and IL-6 cytokine signaling. Consistent with known association of inflammatory pathway activation with progression to TB disease, we found significantly increased expression of the RISK4 (area under the curve [AUC] = 0.734) and PREDICT29 (AUC = 0.736) progression signatures in malnourished individuals.

**Conclusion:**

Malnourished individuals display a peripheral immune response profile reflective of increased inflammation and a concomitant increased expression of risk signatures predicting progression to TB. With validation in prospective clinical cohorts, TB risk biomarkers have the potential to identify malnourished LTBI for targeted therapy.

## Introduction

Tuberculosis (TB) is among the world’s leading causes of death by a single infectious agent (1). Worldwide, over 1.5 million deaths were attributed to TB in 2020, and approximately 1.7 billion people are infected with *Mycobacterium tuberculosis* (Mtb) (1). While most individuals exposed to Mtb develop latent TB infection (LTBI) and remain in that state, 5-10% of infected individuals progress to TB disease (2). Comorbidities such as human immunodeficiency virus (HIV) infection, diabetes mellitus, and alcohol use as well as socioeconomic and environmental factors increase risk of progression from LTBI to TB (1, 3). Malnutrition is an important predisposing factor (3, 4). An estimated 720-811 million individuals worldwide are undernourished and 64% of the global undernourishment is in the 20 countries with 83% of the world’s TB burden. The Food and Agriculture Organization estimated that an additional 118 million individuals experienced hunger in 2020 compared to 2019 likely due to the economic devastation of the COVID-19 pandemic. The population-attributable fraction (PAF) of undernourishment was approximately 19% in 2020 which is greater than both HIV (7.6%) and diabetes (3.1%) (4). In 2016-2020, 24.1% of incident TB in 30 high burden countries was estimated to be attributable to undernutrition. The PAF can be as high as 61.5% in women (5, 6). Observational studies have reported an association between malnutrition and LTBI progression in humans (4–7). A systematic review found a consistent-log linear relationship between body mass index (BMI) and risk of TB disease incidence with every 1kg/m2 decrease being associated with an approximately 14% increase in TB incidence (3). Moreover, approximately 690 million people suffered from malnutrition in 2019, and this number is expected to increase, given the impact of COVID-19 on food security worldwide (8). The World Health Organization (WHO) End TB strategy aims to decrease TB incidence by 90% and TB mortality by 95% by 2035 (9). Addressing malnutrition is crucial to achieving this goal.

There has been a lack of comprehensive studies focused on the mechanism by which malnutrition affects the Mtb immune response. Previous studies using animal models demonstrated effects on innate and adaptive immune responses (7). Mice fed a protein-deficient diet had higher mycobacterial burdens, disorganized granulomas, and lower production of antimycobacterial cytokines NOS2, IFN-γ, and TNF (8). Similarly, guinea pigs fed a protein-deficient diet had poorly formed granulomas and marked reductions in CD4 and CD4 lymphocytes in the blood and spleen (9). Human studies show that malnourished individuals have decreased Th1 (IL-2 and IFN-γ) and proinflammatory (TNF, IL-6, IL-1α, and IL-1β) cytokines and increased Th2 cytokines (IL-4, IL-5, and IL-13) (7, 10, 11). Severe protein-energy malnutrition also mediates atrophy of the thymus and peripheral lymphoid organs, inducing leukopenia, lower CD4/CD8 ratio, more CD4 and CD8 double-negative T cells, and immature T cells in peripheral blood (10, 12). Although these studies have yielded insights pertinent to the immunological consequences of malnutrition, our understanding of human immune response pathways driving progression to TB disease remains considerably limited and requires investigation. In addition, whether immune modulation induced by malnutrition affects the expression of gene signatures predicting risk of progression to TB disease (13–16) also needs inquiry.

In this study, we analyzed whole blood transcriptomic data from malnourished individuals (mal) and control individuals (con) with LTBI in South India with the goal of classifying canonical pathways and gene regulators that are differentially modulated between the two groups. We scored the samples based on published TB risk biomarker gene sets and observed that malnourished individuals had scores that predicted a significantly higher risk for progression to disease compared to those without malnutrition. Identifying malnourished individuals with LTBI who are more likely to progress to TB will enable timely treatment, and also allows one to consider interventions to reduce this risk.

## Methods

### Sample selection

We utilized samples from the Regional Prospective Observational Research in TB (RePORT)-India cohort based at Jawaharlal Institute of Postgraduate Medical Education and Research (JIPMER). The study was conducted in collaboration with Boston Medical Center (BMC), Boston University (BU), JIPMER and Rutgers-New Jersey Medical School. Ethical approval was obtained by the JSAC (46/47/2017; 04/21/2017) and IEC (JIP/IEC JIP/IEC/2017/0149; 07/21/2017) committees of JIPMER and the institutional review boards of BMC/BU (H-35873;12/13/2016) and Rutgers University (Pro20170000497; 5/2/2017).

This study enrolled household contacts (HHC) of newly diagnosed smear-positive, culture-confirmed persons with pulmonary TB identified at National TB Elimination Program clinics. Additional study details have been previously reported (6, 17). Blood was collected from HHC in PaxGene RNA tubes at enrollment. HHC underwent tuberculin skin testing (TST) and were monitored for symptoms of active TB for 24 months; sputum smear and culture were performed on symptomatic individuals, and only LTBI individuals who did not progress to active TB were included in this study.

In addition to demographic characteristics, participant body mass index (BMI) was measured at baseline and categorized into severe malnutrition (BMI < 16 kg/m2), malnutrition (16-18.4 kg/m2), and normal/overweight (>18.4 kg/m2). For this study, individuals with a BMI ≥ 18.4 kg/m2 were referred to as “controls.” In individuals less than 18 years of age, BMI was categorized based on standard deviations relative to the WHO median: children (ages 9-17) whose BMI was more than two standard deviations away from the median for their age were categorized as severely malnourished (6 individuals) and those less than the median were considered malnourished (6 individuals) (18). However, for this study all malnourished and severely malnourished are grouped as “malnourished individuals”. Questionnaires addressed comorbidities that affect host response and TB risk, including HIV, diabetes mellitus, renal failure, alcohol use (using the Alcohol Use Disorders Identification Test [AUDIT-C]), tobacco use, and other parameters (19).

### Sample preparation and analysis

We analyzed RNA-seq data from enrollment PaxGene tubes from a subset of 18 malnourished and 32 control TST- positive (≥5mm) HHCs. PaxGene tubes were sent to MedGenome (Bangalore, India) for processing. RNA was extracted from thawed samples using the PAXgene Blood RNA kit (Cat #762164, Qiagen, Hilden, Germany). Library preparation and sequencing were performed as described previously (17).

Two batches of data were combined: the first batch consisted of 31 samples (15 malnourished and 16 controls; GSE152218), and the second batch consisted of 19 samples (3 malnourished, 18 controls) from our previous study (GSE101705), in addition to samples from individuals with active TB removed after batch correction (17).

### RNA-sequencing data processing

#### QC and alignment

Raw sequencing FASTQ files were assessed for data quality using FastQC (20). Trimmomatic was used to trim the reads (SLIDINGWINDOW:4:20 LEADING:3 TRAILING:3 MINLEN:36) (21). Rsubread was used to align reads to human genome hg38 and to determine expression counts for each gene. Principal component analysis (PCA) of the raw data revealed one outlier LTBI sample that could not be corrected by various methods of normalization or transformation and was subsequently removed before batch correction. Genes with expression count variance less than 20% of the mean variance were excluded from the calculation of differentially expressed genes (DEG).

#### Batch correction

Batch effects created by combining the two batches from GSE152218 and GSE101705 were removed using ComBat-Seq (22). The ComBat-Seq adjusted counts were normalized using a log_2_-counts per million (logCPM) adjustment, and the logCPM values were used for downstream analysis.

#### Differential expression

DEGs between malnourished individuals and control groups were identified using Limma on batch corrected data. The default parameters of Limma were used, with the model design incorporating individuals’ nutrition status. Only protein-coding genes were included to develop a differential pathway list of malnourished individuals vs. control individuals.

#### Dimension reduction

PCA and t-distributed Stochastic Neighbor Embedding (tSNE) were used to reduce the dimensionality of the data and project the data into two dimensions (using the PlotPCA function from the DESeq2 and Rtsne R packages, respectively).

#### Pathway enrichment analysis

Due to the low number of DEGs with an FDR > 0.05, we used the top 2923 genes with a p-value <0.05 identified between malnourished individuals and controls to identify potential biologically relevant pathways. The gene list was analyzed with Qiagen’s Ingenuity Pathway Analysis (IPA), using recommended standards (23). The activation z-score provided a statistical measure of the direction of gene regulation (a positive z-score indicates predicted activation, and a negative z-score indicates predicted inhibition). To control for false positives, we used a z-score of 2.0 as a cutoff and p<0.01 to identify canonical pathways, and a lower than normal p-value of p<0.001 for upstream and master regulators.

### TBSignatureProfiler platform

The TBSignatureProfiler was used to profile signatures; it contains functions for analyzing gene lists from pathways or signatures to determine the predictive value (24). Scoring methods used here were Gene Set Variance Analysis (GSVA) and single sample Gene Set Enrichment Analysis (ssGSEA). Heatmaps, boxplots, receiver-operating characteristic (ROC) curve, and area-under the ROC-curve (AUC) were calculated and depicted using the functions within the TBSignatureProfiler. Bootstrapping was used to iteratively calculate AUC values using leave-one-out cross-validation to obtain mean AUCs and 95% confidence intervals (CI) for 100 repeats for each signature.

Over fifty previously published TB signatures were available in the TBSignatureProfiler at the time of this study. However, only those related to predicting risk of developing TB were used for this study: the Suliman 4-gene signature (denoted as RISK4) derived from a study of African, HIV-uninfected HHCs for prediction of TB progression up to two years before TB onset (13); the Sweeney 3-gene signature (SWEENEY3) was derived from a meta-analysis using 14 datasets and was reported to separate TB from other diseases (14); the Zak 16-gene signature (ACS COR), derived from a South African adolescent cohort study, described a prospective signature of TB risk up to 12 months preceding TB diagnosis (16); and the Leong 29-gene signature (denoted as PREDICT29), which was derived in an African cohort and validated in a Brazilian cohort and predicts risk of progression/reactivation in exposed individuals at least five years before TB disease development (15).

We generated mean AUC values for the predictive performance of these biomarkers in their ability to distinguish between malnourished individuals and control samples in our cohort. We also used the control group ssGSEA scores to generate an empirical “high risk” cutoff for each signature and then observed the proportion of malnourished individuals with scores above that cutoff. We considered cutoffs at the 75th (and 90th) percentiles of the control group scores for this comparison. In addition, we calculated the Youden’s index using the cutpointR package in R (calculated by adding the sensitivity and specificity of a ROC curve and subtracting 1 with a higher index representing better diagnostic ability) (25).

### Data availability and accessibility

Processed data were analyzed using R version 4.0.1, and the code and files are available on GitHub at https://github.com/avanvalken/LTBI_malnutrition_RNAseq. Processed and raw RNA-seq data are available. The datasets presented in this study can be found in online repositories. The names of the repository/repositories and accession numbers are: https://www.ncbi.nlm.nih.gov/geo/, GSE152218; and https://www.ncbi.nlm.nih.gov/geo/, GSE101705.

## Results

### Demographics

Of 50 HHCs with LTBI, 18 were malnourished individuals, and 32 were controls. Overall, 24 (48.0%) were male, and the median age was 26.5 years (range 9-80) (**Table 1**). Within the malnourished individuals and control groups, 9 (50.0%) and 15 (46.9%) were male, and the median ages were 13 years (range 9-35) and 37 years (range 13-80), respectively. One malnourished individual (5.6%) and five controls (15.6%) reported alcohol use. No malnourished participants reported tobacco use, and one malnourished participant reported diabetes mellitus (5.6%). Of the control group, one (2.94%) reported smoking, and 1 (3.13%) diabetes mellitus. No statistically significant differences were found between malnourished individuals and control groups for tobacco use, alcohol use, diabetes, or sex, although age was significantly younger in the malnourished group (p=8.94e-9).

**Table 1:** Demographic characteristics.

	Malnourished	Controls	Total	P value
	*n* = 18	*n* = 32	*n* = 50	
Median age, years (range)	13 (9-35)	37 (12-80)	26.5 (9-80)	8.94e-9
Sex, *n* (%)				
Male	9 (50.0)	15 (46.9)	24 (48.0)	1.0
Female	9 (50.0)	17 (53.1)	26 (52.0)	
Smoking, *n* (%)				
Ever	0	1 (2.94)	1 (2.0)	1.0
Never	18 (100)	33 (97.1	49 (98.0)	
Alcohol, *n* (%)				
Ever	1 (5.6)	5 (15.6)	6 (12.0)	0.399
Never	17 (94.4)	27 (84.4)	44 (88.0)	
Diabetes, *n* (%)				
Yes	3 (16.7)	1 (3.13)	4 (8.0)	0.127
No	15 (83.3)	31 (96.9)	46 (92.0)	
Relation to PLWTB, *n* (%)				3.41e-4
Sibling	3 (16.7)	3 (9.4)	6 (12.0)	
Parent	0	4 (12.5)	4 (8.0)	
Child	12 (66.7)	10 (31.3)	22 (44.0)	
Spouse	0	14 (43.8)	14 (28.0)	
Other	3 (16.7)	1 (3.1)	4 (8.0)	

Table depicting demographic characteristics of HHCs with LTBI.

PLWTB, person living with TB. P-values were calculated by a Welch’s t-test for ages between malnourished individuals and controls, and Fisher’s exact test for sex, smoking, alcohol, diabetes, and relation to PLWTB.

### Differential gene expression and dimension reduction analyses

**Figure 1:**
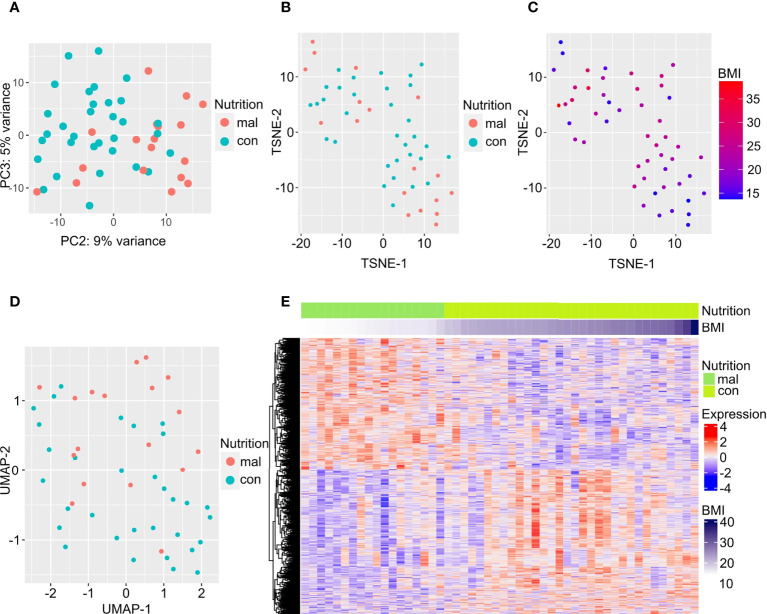
Differentially expressed genes separate individuals with LTBI who are malnourished from controls. Dimension reduction by PCA **(A)**, tSNE **(B, C)**, and UMAP **(D)** of RNA-sequencing data are plotted here, with the points in C colored by BMI. The top 500 DEGs (by least adjusted p-value) are depicted in the heatmap; columns are organized by BMI of individuals with LTBI from lowest to highest **(E)**.

We first examined gene expression differences between the malnourished individuals and controls in our dataset. We used unsupervised computational methods and supervised methods to identify differences between the malnourished and control groups. Unsupervised dimension reduction with PCA (**Figure 1A**), tSNE (**Figure 1B**) and UMAP (**Figure 1D**) demonstrated that the majority of malnourished and controls segregated into two groups. However, a few individuals from both groups segregated inaccurately based on their nutritional status. This clustering pattern was further confirmed by a tSNE plot colored by BMI (**Figure 1C**), and a heatmap of the top 500 DEGs by lowest adjusted p-value (with 288 upregulated and 212 downregulated genes) depicting individuals with intermediate BMI expressing a gene expression profile similar to that of individuals with a BMI<18.4 kg/m2 (**Figure 1E**). As shown in **Figure 1E**, the gene expression patterns show a graded change with increasing BMI, indicating that there is a correlation between BMI and gene expression pattern. A volcano plot depicting the differentially expressed genes between the malnourished and controls with LTBI is depicted in **Supplementary Figure 1**. The top DEGs (ranked by lowest adjusted p-value) are highlighted. Genes upregulated in controls included *EDA* involved in cell growth (26) and *CTSE* which encodes for an aspartic proteinase implicated in antigen processing within the class II MHC pathway (27). Several of the top DEGs upregulated in the malnourished group such as *CD27*, *CD38* and *CD7* are molecules regulating T cell activation. CD27 is a co-stimulatory molecule belonging to the tumor necrosis factor receptor (TNFR) family (28, 29) CD7 is another co-stimulatory molecule that is involved in T and NK cell activation (30–32). CD38 is a cell surface glycoprotein with receptor and enzymatic functions (33). The NAD^+^ glycohydrolase activity of CD38 promotes T cell activation and proliferation (34).

### Malnutrition is associated with increased inflammation and immunomodulation

**Figure 2:**
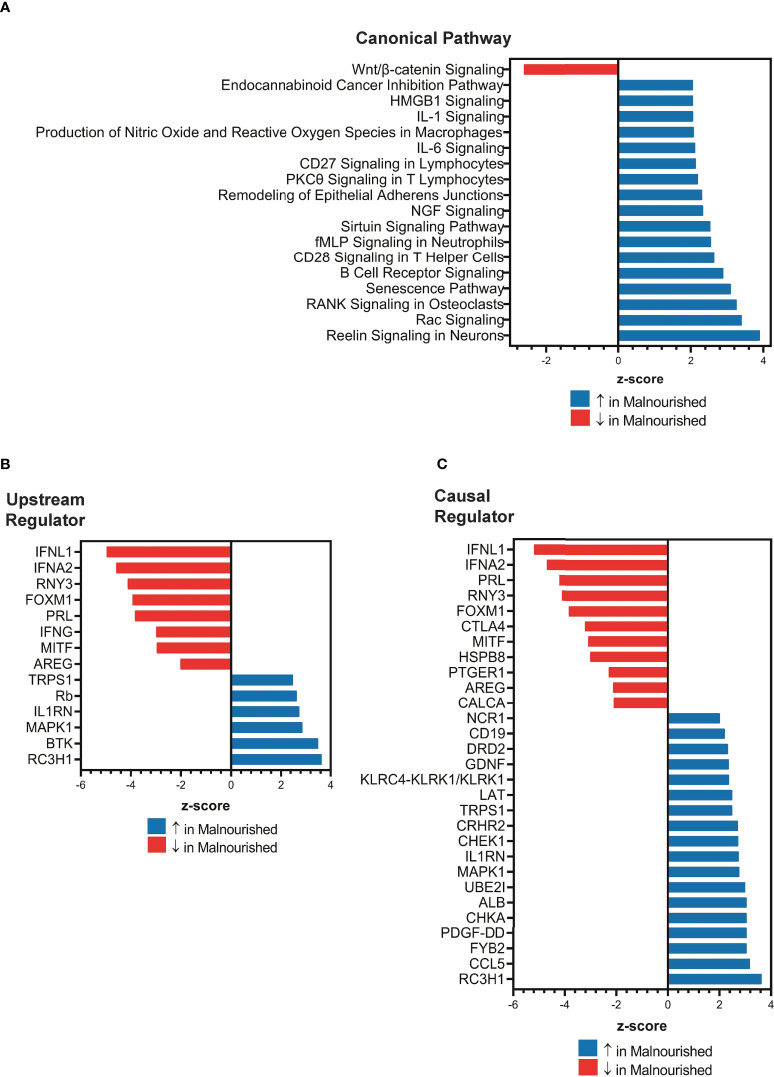
Increased inflammation and immune regulation pathways in malnourished individuals with latent TB infection. IPA of DEGs among malnourished individuals and control individuals showing top canonical pathways [p < 0.01; **(A)**], upstream regulators [p < 0.001; **(B)**], and causal regulators (p < 0.001; **(C)**). Pathways and regulators in blue represent upregulation and those in red represent downregulation in malnourished individuals.

Next, we used IPA to conduct an unbiased analysis of DEGs to identify activated or inhibited immune response pathways and gene regulators. The top upregulated canonical pathways in malnourished included senescence, neutrophil activation (fMLP signaling), T-cell activation (CD28 signaling, PKC signaling, T-cell receptor signaling molecules), B cell receptor signaling, proinflammatory cytokine signaling (IL-1 and IL-6), HMGB1 signaling, and Rac signaling (**Figure 2A**). Of note, Wnt/β-catenin signaling was the only downregulated pathway in malnourished (**Figure 2A**).

Upstream regulator analysis indicated that the immunomodulatory genes PRL (Prolactin, growth hormone and cytokine), IFNL1 (type III interferon), RNY3 (small RNA Y3), AREG (Amphiregulin, growth factor), IFNA2 (type I interferon), and IFN-γ (type II interferon) were inhibited in malnourished individuals (**Figure 2B**). In contrast, IL1RN (IL-1 receptor antagonist) and MAPK1 (MAP kinase 1) were among the genes that were predicted to be activated in malnourished (**Figure 2B**). We conducted causal network analysis to identify the predictive activity patterns of causative master regulators upstream of targets in the DEG dataset. Most genes in the upstream regulator analysis had similar activation and inhibition states, except for IFN-γ, which was not among the top master regulators (**Figure 2C**). In addition, NCR1 (NK cell activating receptor), CD19 (B-lymphocyte surface antigen B4), LAT (linker of activated T cells), and CCL5 (C-C Motif Chemokine Ligand 5) were predicted to be activated, while CTLA4 (a negative regulator of T-cell activation) was predicted to be inhibited in malnourished individuals (**Figure 2C**).

### Gene sets within published TB risk biomarkers are significantly increased in malnourished individuals

In Mtb infected individuals, sequential increase in inflammatory gene expression precedes diagnosis of tuberculosis (35). We therefore hypothesized that the increased inflammation present in the malnourished group could be associated with an increased risk of progression to TB. To test the hypothesis, we evaluated four published TB risk signatures (13–16).

**Figure 3:**
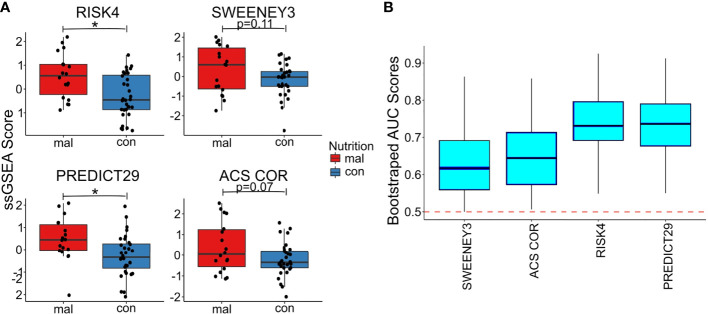
Malnourished individuals with LTBI demonstrate a higher risk of TB progression than controls with LTBI. Accuracy of TB risk signatures in predicting differences between malnourished individuals and control groups as depicted by boxplots showing ssGSEA scores [RISK4, p=0.0003; PREDICT29, p = 0.0038; ACS COR, p = 0.076; and SWEENEY3, p = 0.10; **(A)**] and bootstrapped upper and lower AUC scores and means [RISK4, AUC = 0.73; PREDICT29, AUC = 0.74; ACS COR, AUC = 0.63; and SWEENEY3, AUC = 0.61; **(B)**]. * significant p value.

We used the TBSignatureProfiler to evaluate the expression of the 4 TB risk signatures. All signatures scored malnourished individuals higher than controls; RISK4 (p=0.0035, AUC=0.734) and PREDICT29 (p=0.012, AUC=0.736) were the most significantly increased in the malnourished group (**Figures 3A, B**, **Supplementary Figure 2**) and demonstrated the highest AUC scores. Although there are common inflammatory genes such as Guanylate Binding Protein (GBP) 5 and SEPTIN4 among RISK4, SWEENEY3, and ZAK16, there is no overlap of genes from the other biomarkers with PREDICT29 (**Supplementary Figure 1**). Instead, PREDICT29 includes genes associated with early innate immune response, including SH2D1B (SH2 Domain containing 1B), CTSA (Cathepsin A), SPSB1 (SplA/Ryanodine Receptor Domain And SOCS Box Containing 1), IL31RA (Interleukin 31 Receptor A) and HM13 (Histocompatibility Minor 13) (15).

We also used the control risk scores to generate an empirical “high risk” score cutoff, as determined by the 75^th^ (and 90^th^) percentiles of the risk scores from the control individuals. The malnourished group had elevated risk scores for all signatures, as a higher proportion of the malnourished individuals were above the cutoff than was expected (i.e., expected 25% or 10%). In particular, the ssGSEA scores using the PREDICT29 signature ranked 55.6% (33.3%) of the individuals in the malnourished group above the 75^th^ (90^th^) percentile of the control group. The other signatures also showed large increases in risk scores above the high-risk cutoff: 50.0% (38.9%) of malnourished individuals scored above the 75^th^ (90^th^) percentile of control individuals using the RISK4 signature, 55.6% (38.9%) using the SWEENEY3 signature, and 44.4% (33.3%) using the ACS COR signature (**Table 2**). Using Youden’s index, PREDICT29 was found to have an index of 0.44, followed by RISK4’s 0.38, SWEENEY3’s 0.37, and ACS COR’s 0.32. These results demonstrate that the differences in gene expression led to an increase in enrichment scores of TB risk signatures within the malnourished group.

**Table 2:** Percent of malnourished individuals above percentile cutoff of controls.

TB Risk Signatures	75th Percentile (%)	90th Percentile (%)
ACS COR	44.4	33.3
SWEENEY3	50.0	38.9
RISK4	55.6	38.9
PREDICT29	55.6	33.3

Table depicting the percentage of malnourished individuals with higher ssGSEA scores than the 75^th^ or 90^th^ percentile cutoffs of the controls for each TB risk signature.

## Discussion

RNA-sequencing data from malnourished individuals and controls with LTBI revealed that the canonical pathways, predicted activation and inhibition patterns of the upstream regulators, and master regulators showed an overall dysregulated immune response in malnourished individuals. Specifically, we observed increased inflammatory response accompanied by suppression of immunoregulation in malnourished individuals. Furthermore, increased expression of gene sets from published TB risk biomarkers indicated that malnourished individuals were associated with an increased risk of TB.

In malnourished individuals, immune response pathways involved in neutrophil activation (fMLP signaling), T-cell activation (CD28 signaling, PKC signaling, T-cell receptor signaling molecules), and proinflammatory cytokine signaling (IL-1 and IL-6) were upregulated. In particular, we found HMGB1 signaling pathway was upregulated in malnourished individuals. HMGB1 is a damage-associated molecular pattern molecule and promotes inflammation when released from damaged cells (36) supporting increased inflammation in malnourished individuals. We also found upregulation of the senescence signaling pathway in malnourished individuals which could be contributing to the enhanced inflammation associated with malnutrition. Senescent cells are not dormant and actively secrete proteins which is referred to senescence-associated secretory phenotype (SASP) (37). The SASP includes a plethora of cytokines and chemokines (38). Of note SASP factors secreted by senescent cells also induce CD38 expression (39), which expression was significantly upregulated in the malnourished group. T regulatory cells and myeloid-derived suppressor cell derived from CD38KO mice have enhanced cytokine production and are functionally less suppressive. In addition, inhibition of CD38 increases glutaminolysis in T cells and enhances their anti-tumor activity (40). It is possible that although there is an overall inflammatory response in the malnourished group, the increased CD38 expression could nonetheless affect antigen-specific T cell effector functions. Future studies should investigate the impact of malnutrition on T cell immunometabolism and subsequent functional activity.

Interestingly, the only significantly downregulated pathway in malnourished individuals was Wnt/β-catenin signaling. Besides playing a critical role in cell growth, homeostasis, and differentiation, Wnt/β-catenin signaling can also modulate the immune response (41) by repressing NFκB activation and curtailing the production of proinflammatory cytokines from cells in response to a number of stimuli (42). Another mechanism that Wnt/β-catenin signaling employs to limit inflammation is by biasing dendritic cells to a tolerogenic state (43). Furthermore, in mouse models of TB an inverse correlation of Wnt/β-catenin signaling and inflammation has been reported (44, 45). Together, these findings suggest that in malnourished individuals, inflammation is further promoted by downmodulation of the Wnt/β-catenin signaling.

IPA revealed several inhibited immunomodulatory regulators. Among these immunomodulatory regulators, IFNL1 is of significance since it is anti-inflammatory and has been reported to curb inflammation *via* suppression of neutrophil infiltration (46), IL-1β production (46), and also *via* non-translational inhibition of ROS production and degranulation of neutrophils (47). Another immunomodulator of interest is AREG which promotes CD4+ Treg cell-mediated suppression of localized immune responses (48). Despite increased activation of inflammatory pathways in malnourished individuals, however, we observed a predicted downregulation of IFN-γ in this group. Data from a previous study showing diminished induction of cytokines in malnourished individuals in response to Mtb antigens (11) leads us to posit that in malnourished individuals, there may be attrition of Mtb antigen-specific T cells which places them at higher risk of progression to TB. The increased expression of TB risk signatures supports our hypothesis.

The study had the following limitations. Although the individuals included in this study did not develop active TB disease for at least two years after blood samples were taken, it is likely the effects of malnutrition on immune pathways leads to reactivation of LTBI at a later time. Furthermore, malnourished individuals are likely to experience deficiencies in several nutrients that impact the immune system, such as vitamins A and D, E and others. We did not directly address the contribution of individual micronutrient deficiencies toward TB risk and expression of risk signatures. This is important since there is accumulating evidence that vitamin deficiencies (particularly vitamins A, D, and E) increase the risk of developing active TB. Studies demonstrate that vitamin A deficiency might increase TB risk up to 10-fold. A cohort study of HIV-uninfected Peruvians found that serum retinol <0.70 μmol/L was associated with increased TB risk in close contacts after adjusting for confounders (49). Among 332 HIV-infected individuals, serum retinol <0.7umol/L was associated with an adjusted hazard ratio (aHR) of 5.33 (95%CI 1.54-18.43) for developing TB compared to those with normal vitamin A levels (50). Analysis of baseline samples of a longitudinal cohort study nested within a randomized clinical trial among HIV+ adults in Haiti found that vitamin A deficiency was a good predicter of incident tuberculosis (51). Higher levels of pro-vitamin A carotenoids (that are metabolized intracellularly to vitamin A) in conjunction with low IL-18 were associated with reduced hazard of incident TB among 290 HIV-infected individuals (aHR 0.48; 95% CI 0.26–0.87) (52). Vitamin D deficiency (<20 ng/mL) is associated with incident TB in contacts of TB patients and HIV-infected individuals (HR 2.89 [95%CI 1.31-7.41] and aHR 3.66 [95% CI 1.16-11.51] respectively) (50, 53) and separately vitamin D (25(OH)D) <75nmol/l associated with an OR of 6.5 (95%CI 1.8-23.5) for increased risk of TB among contacts of TB cases in Greenland (54). A meta-analysis found that vitamin D levels <12.5 nmol/L are associated with a pooled OR for TB risk of 4.6 (95% CI 2.2 - 9.4) (55). Another meta-analysis of data from 3,544 participants found that severe vitamin D deficiency had an OR of 2.05 (95%CI 0.87–4.87) for TB risk overall and 4.28 (95%CI 0.85–21.45; p=.08) among HIV-infected individuals (56). That same cohort study from Peru showed that having the lowest tertile (compared to highest) of δ-Tocopherol (vitamin E) was associated with a 2.29-fold increased TB risk in close contacts after adjusting for confounders (95%CI 1.29-4.09) (57).

Host genetic studies of tuberculosis suggest that besides co-morbidities such as malnutrition, genetic factors may also influence susceptibility to pulmonary tuberculosis (58, 59). These genetic association studies focused on candidate genes and reported that sequence variants in several immunity-related genes influenced tuberculosis susceptibility (60). However, most of these studies were under-powered and conducted in different ethnic populations and so it has been difficult to reach a consensus on tuberculosis disease susceptibility genes (60, 61) Findings from genome-wide association studies of pulmonary tuberculosis have also not yielded clear data in terms of whether common variants may have an effect on individual susceptibility to adult pulmonary tuberculosis. Although, a meta-analyses study of genetic association with tuberculosis risk found 9 variants in 9 genes showing strong cumulative evidence for significant association with risk of TB (62). Our study cohort consists mainly of children and the association of increased susceptibility to tuberculosis in children with mendelian inborn errors of immunity (63, 64) suggests that the influence of underlying genetic background should be considered in our malnourished study population. Furthermore, host responses to mycobacteria can be influenced by age-associated differences. For example, macrophages from infants have delayed maturation of toll-like receptors over the first year of life (65, 66), reduced phagocytic capacity for Mtb (67) and reduced cytokine production (68) in comparison to adult macrophages. At the adaptive T cell level, distinct methylation patterns exist in CD4^+^ naive T cells between cord blood and adult peripheral blood which results in differences in IFN-γ production and T cell effector functions (69). Whether the compromised innate and T cell functions in the pediatric population, together with increased numbers of regulatory T cells (70) would lead to impaired Mtb immunity and increased risk of progression to TB needs investigation.

Despite these limitations, our transcriptomic study suggests that malnourished individuals with LTBI are a particularly vulnerable population predisposed to increased risk of progression to TB. Using a combination of *in-vitro* models and animal models, future studies should focus on validating and evaluating the mechanistic basis of the increased inflammatory immune responses in malnutrition. In addition, studies investigating the immunological outcomes of nutritional interventions are needed to determine whether large-scale nutritional supplementation should be considered to decrease TB risk. TB risk biomarkers could be effectively employed to risk-stratify and identify malnourished individuals with LTBI for preventive therapy or nutritional interventions. Insights from these studies will be integral to the End TB strategy.

## Data availability statement

The datasets presented in this study can be found in online repositories. The names of the repository/repositories and accession number(s) can be found below: https://www.ncbi.nlm.nih.gov/geo/, GSE152218; https://www.ncbi.nlm.nih.gov/geo/, GSE101705.

## Ethics statement

The studies involving human participants were reviewed and approved by Institutional Review Board- Boston University Medical Campus, Rutgers University and JSAC and IEC committees of JIPMER. Written informed consent to participate in this study was provided by the participants’ legal guardian/next of kin.

## Author contributions

AV and VK acquired the data and wrote the first draft of the manuscript. WJ, NH, and PS were responsible for the conception and design of the study. SS, SL, NJ, CH, and JE contributed to the design of the study. CC, SP, SK, and PBN wrote sections of the manuscript. All authors contributed to manuscript revision, read, and approved the submitted version.

## Funding

This work was supported by Award No. USB-31150-XX-13 of the US Civilian Research & Development Foundation (CRDF Global) and the National Science Foundation under Cooperative Agreement No. OISE-9531011 with Federal funds from the Government of India’s (GOI) Department of Biotechnology (DBT), the United States National Institutes of Health (NIH), National Institute of Allergy and Infectious Diseases (NIAID), Office of AIDS Research (OAR), and distributed in part by CRDF Global; Warren Alpert Foundation; Boston University School of Medicine; and National Institutes of Health (R01GM127430 and R21AI154387). The contents of this publication are solely the responsibility of the authors and do not represent the official views of the DBT, the NIH, or CRDF Global.

## Acknowledgments

We are grateful to the participants and their families, without whom the study would not have been possible. We also acknowledge the contributions of the field teams at JIPMER who have helped carry out these studies and our prior study coordinators Rachel Kubiak and Jane Pleskunas.

## Conflict of interest

The authors declare that the research was conducted in the absence of any commercial or financial relationships that could be construed as a potential conflict of interest.

## Publisher’s note

All claims expressed in this article are solely those of the authors and do not necessarily represent those of their affiliated organizations, or those of the publisher, the editors and the reviewers. Any product that may be evaluated in this article, or claim that may be made by its manufacturer, is not guaranteed or endorsed by the publisher.

## Supplementary material

Supplementary Figure 1 A Volcano plot of differentially expressed genes between the malnourished and controls suggests differential immune regulation. A volcano plot depicting the differentially expressed genes between the malnourished and controls with LTBI is depicted here, with the top 15 genes by lowest p-value labeled. Differential expression is relative to controls, with blue signifying downregulation in controls, and red signifying upregulation in controls, with an adjusted p-value of < 0.1. Non-significant genes were colored as black.

Supplementary Figure 2 Differential expression patterns of all genes within each risk signature. Gene enrichment of TB risk signatures is scored by ssGSEA and GSVA; heatmaps for each signature are depicted. RISK4 **(A)**, SWEENEY3 **(B)**, PREDICT29 **(C)**, and ACS COR **(D)** are each depicted in their respective panels.
