# Corrigendum: Malnutrition leads to increased inflammation and expression of tuberculosis risk signatures in recently exposed household contacts of pulmonary tuberculosis

**DOI:** 10.3389/fimmu.2022.1064883

**Published:** 2022-10-20

**Authors:** Arthur VanValkenburg, Vaishnavi Kaipilyawar, Sonali Sarkar, Subitha Lakshminarayanan, Chelsie Cintron, Senbagavalli Prakash Babu, Selby Knudsen, Noyal Mariya Joseph, C. Robert Horsburgh, Pranay Sinha, Jerrold J. Ellner, Prakash Babu Narasimhan, William Evan Johnson, Natasha S. Hochberg, Padmini Salgame

**Affiliations:** ^1^ Division of Computational Biomedicine, Boston University School of Medicine, Boston, MA, United States; ^2^ Bioinformatics Program, Boston University, Boston, MA, United States; ^3^ Department of Medicine, Center for Emerging Pathogens, Rutgers-New Jersey Medical School, Newark, NJ, United States; ^4^ Department of Preventive and Social Medicine, Jawaharlal Institute of Postgraduate Medical Education and Research, Puducherry, India; ^5^ Department of Medicine, Boston Medical Center, Boston, MA, United States; ^6^ Department of Microbiology, Jawaharlal Institute of Postgraduate Medical Education and Research, Puducherry, India; ^7^ Section of Infectious Diseases, Boston University School of Medicine, Boston, MA, United States; ^8^ Department of Epidemiology, Boston University School of Public Health, Boston, MA, United States; ^9^ Department of Clinical Immunology, Jawaharlal Institute of Postgraduate Medical Education and Research, Puducherry, India

**Keywords:** tuberculosis, malnutrition, TB biomarkers, inflammation, immunoregulation

In the published article, there was an error in the author list, and author Pranay Sinha was erroneously excluded. The corrected author list appears below.

Arthur VanValkenburg^1,2†^, Vaishnavi Kaipilyawar^3†^, Sonali Sarkar^4^, Subitha Lakshminarayanan^4^, Chelsie Cintron^5^, Senbagavalli Prakash Babu^4^, Selby Knudsen^5^,Noyal Mariya Joseph^6^, C. Robert Horsburgh^7,8^, Pranay Sinha^5^, Jerrold J. Ellner^3^, Prakash Babu Narasimhan^9^,

W. Evan Johnson^1,2‡^, Natasha S. Hochberg^5,7,8‡^ and Padmini Salgame^3*‡^

The authors apologize for this error and state that this does not change the scientific conclusions of the article in any way. The original article has been updated.

## Publisher’s note

All claims expressed in this article are solely those of the authors and do not necessarily represent those of their affiliated organizations, or those of the publisher, the editors and the reviewers. Any product that may be evaluated in this article, or claim that may be made by its manufacturer, is not guaranteed or endorsed by the publisher.

