# Supplementary figures and images for: Malnutrition leads to increased inflammation and expression of tuberculosis risk signatures in recently exposed household contacts of pulmonary tuberculosis
Source: Front Immunol. 2022 Sep 28;13:1011166. doi: 10.3389/fimmu.2022.1011166 (PMC9554585; doi:10.3389/fimmu.2022.1011166)

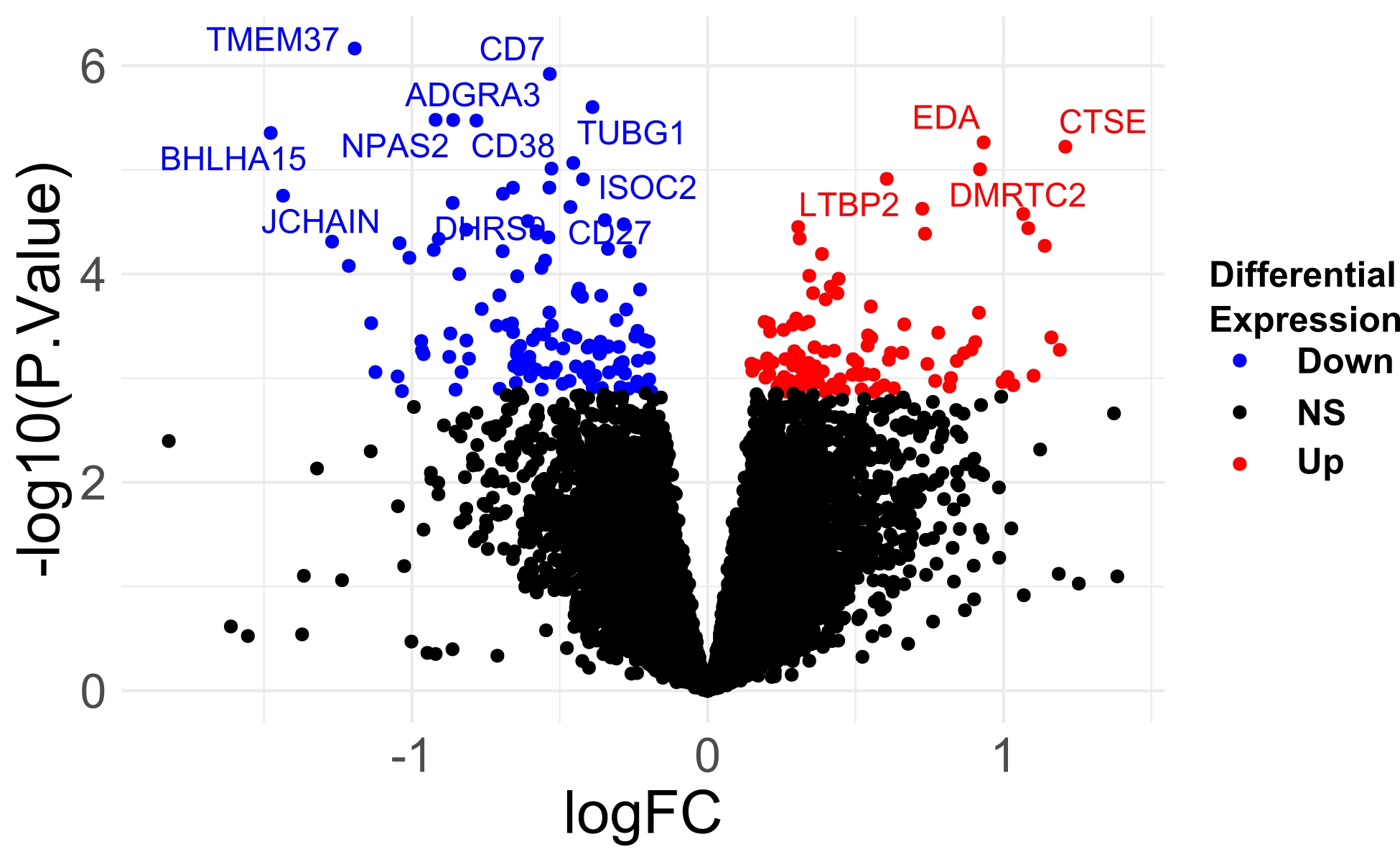

Supplement: Supplementary Figure 1 — A Volcano plot of differentially expressed genes between the malnourished and controls suggests differential immune regulation. A volcano plot depicting the differentially expressed genes between the malnourished and controls with LTBI is depicted here, with the top 15 genes by lowest p-value labeled. Differential expression is relative to controls, with blue signifying downregulation in controls, and red signifying upregulation in controls, with an adjusted p-value of < 0.1. Non-significant genes were colored as black. [file Image_1.jpeg]

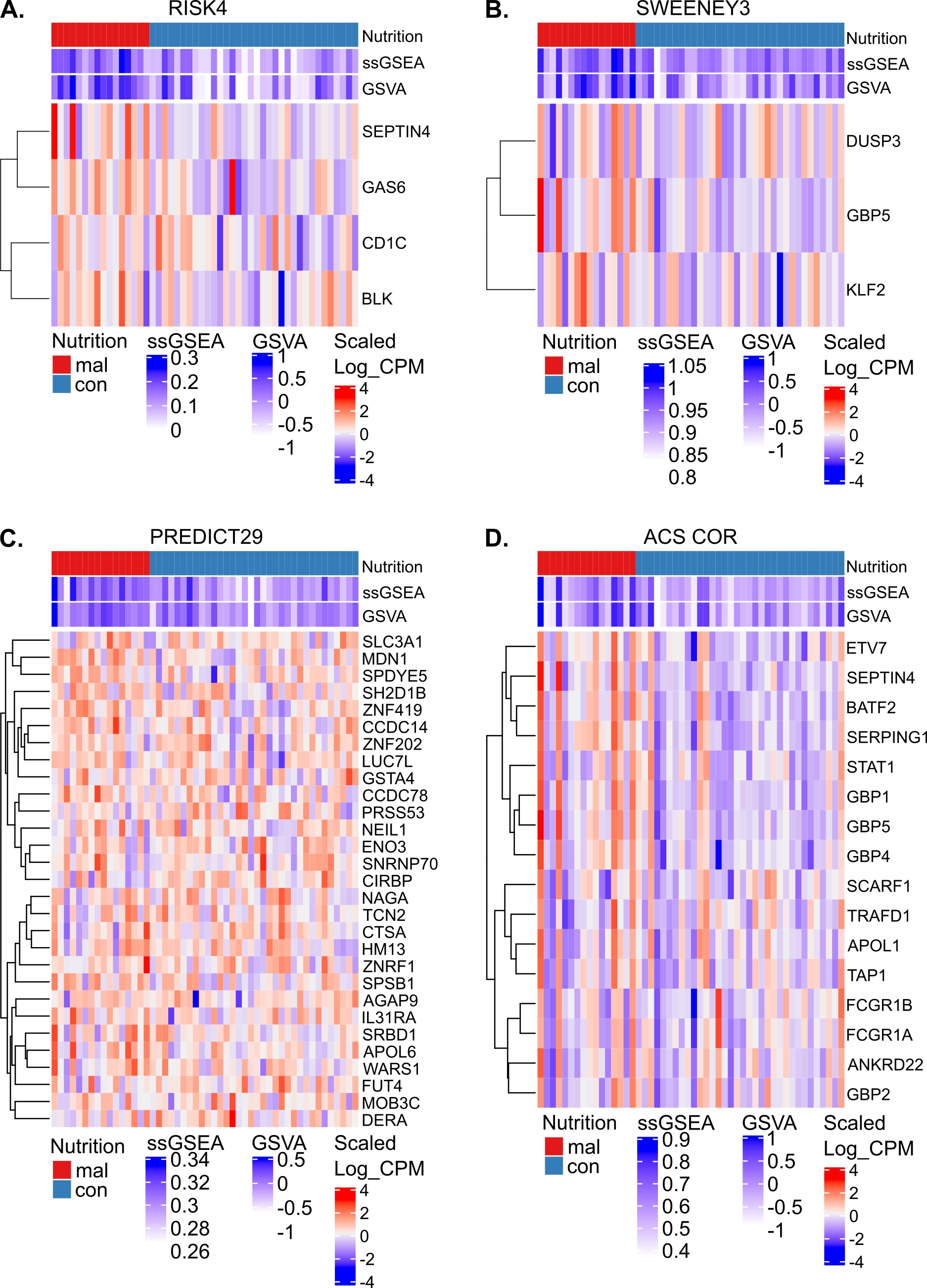

Supplement: Supplementary Figure 2 — Differential expression patterns of all genes within each risk signature. Gene enrichment of TB risk signatures is scored by ssGSEA and GSVA; heatmaps for each signature are depicted. RISK4 (A), SWEENEY3 (B), PREDICT29 (C), and ACS COR (D) are each depicted in their respective panels. [file Image_2.jpeg]
